# Cystitis Glandularis: A 10-Year Institutional Experience and Review of the Literature

**DOI:** 10.7759/cureus.98588

**Published:** 2025-12-06

**Authors:** Nawal Khan, Yasmin Abu Ghanem, Wen Ng, Rajesh Nair, Elsie Mensah, Ramesh Thurairaja, Muhammad S Khan

**Affiliations:** 1 Urology, Guy's and St Thomas' NHS Foundation Trust, London, GBR; 2 Pathology, Guy's and St Thomas' NHS Foundation Trust, London, GBR; 3 Urology, King's College London, Guy's and St. Thomas' Hospitals, King's Health Partners, London, GBR

**Keywords:** adenocarcinoma, bladder, cystitis cystica, cystitis glandularis, intestinal metaplasia, urology

## Abstract

Aims

Cystitis glandularis (CG) and cystitis cystica et glandularis (CCEG) are benign proliferative lesions of the bladder that may closely mimic carcinoma on imaging, cystoscopy, and histology. Their natural history and malignant potential remain uncertain, which can make management challenging for urologists. This study aimed to analyze the clinical behavior and malignant potential of CG and CCEG by reviewing a decade of institutional experience alongside current literature.

Methods

This retrospective observational review of all patients diagnosed with CG, cystitis cystica, or CCEG at Guy's and St Thomas' Hospital was conducted over a 10-year period. Demographic data (including age, sex, ethnicity, and relevant clinical history), presenting symptoms, imaging and cystoscopic findings, histopathology, treatment, and follow-up information were collected. The published literature was reviewed with a focus on histological features, immunohistochemistry, molecular findings, and the possible association with malignancy.

Results

Sixty-seven patients were identified. Median age at diagnosis was 61 years (range: 39-84 years), and 57 (85.1%) were male. The most common presentations were lower urinary tract symptoms (LUTS; 28.4%), visible hematuria (20.9%), and non-visible hematuria (16.4%). Cystitis glandularis was present in 30 (44.8%) patients, and intestinal-type metaplasia in 16 (23.9%) patients. Cystitis cystica was found in 53 cases (79.1%), and was associated with urothelial carcinoma in six (9.0%) patients. Lesions most frequently involved the bladder neck or trigone (62.7%). Median follow-up was 9.2 years (range: 2.2-17.8 years). No patient in this series developed de novo carcinoma arising from CG or CCEG during surveillance. Two patients with extensive trigonal involvement and upper tract obstruction underwent robot-assisted bilateral ureteric reimplantation.

Conclusion

CG and CCEG are usually benign conditions but can be difficult to distinguish from primary adenocarcinoma of the bladder, especially when intestinal-type metaplasia or mucus extravasation is present. The intestinal variant, particularly when extensive or associated with dysplasia, has been proposed as a potential premalignant lesion. Molecular studies show some overlap in gene expression profiles between CG and urothelial carcinoma, but clinical series have not consistently demonstrated an increased risk of progression. Management is centered on relief of underlying irritation, endoscopic resection of mass-like lesions, and surveillance in selected cases. In patients with extensive disease causing obstruction, reconstructive procedures, such as ureteric reimplantation or, rarely, cystectomy, may be required.

## Introduction

Benign epithelial proliferations of the urinary bladder sometimes resemble malignant disease, both endoscopically and histologically. Among these, cystitis glandularis (CG) and cystitis cystica et glandularis (CCEG) are relatively common but frequently misunderstood lesions. They may present incidentally, or with non-specific lower urinary tract symptoms, but their appearance can be highly suggestive of carcinoma. This, combined with uncertainty about their natural history, can lead to considerable diagnostic and therapeutic dilemmas.

CG arises from von Brunn's nests, which are clusters of normal urothelium that invaginate into the lamina propria [1]. These nests can undergo cavitation and glandular metaplasia. Two main patterns are recognized. In conventional CG, glands are lined by cuboidal to low columnar epithelium and are often surrounded by residual urothelial cells [2]. In intestinal-type CG, the glandular epithelium is composed of tall columnar mucin-secreting cells, frequently with goblet cells and, occasionally, Paneth cells [2]. This intestinal phenotype closely resembles colonic mucosa and can be very difficult to distinguish from primary adenocarcinoma of the bladder.

CG often coexists with cystitis cystica, in which von Brunn's nests become cystically dilated within the lamina propria [2]. When both cystic and glandular elements are present, the lesion is termed cystitis cystica et glandularis (CCEG). CCEG with intestinal metaplasia (sometimes abbreviated CCGIM) is characterized histologically by the absence of cytological atypia, necrosis, signet-ring cells, and atypical mitoses, and is therefore regarded as a benign lesion [3]. However, extensive glands, mucus extravasation, and involvement of deeper muscle bundles can be mistaken for invasive adenocarcinoma.

The etiology of CG and CCEG is thought to relate to chronic irritation or inflammation of the bladder mucosa. Reported associations include recurrent urinary tract infections, bladder stones, bladder outlet obstruction, long-term indwelling catheters, bladder exstrophy, neurogenic bladder, and pelvic lipomatosis [4-10]. Hormonal influences, allergic phenomena, and IgA-mediated immune mechanisms have also been suggested [11]. CG has been reported in association with 14-67% of non‑urachal adenocarcinomas of the bladder, although this coexistence does not confirm a precursor relationship and may instead reflect shared inflammatory pathways [12].

Clinically, patients may be entirely asymptomatic, with lesions detected incidentally on cystoscopy or imaging undertaken for other reasons. When symptoms occur, they are usually non-specific and include lower urinary tract symptoms (LUTS), visible or non-visible hematuria, and suprapubic or flank pain [13]. Extensive involvement of the trigone or bladder neck can cause bladder outlet obstruction or ureteric obstruction with hydroureteronephrosis and, in severe cases, renal impairment [5,14-17].

Because of the overlap in appearance with malignant lesions, diagnosis usually requires cystoscopic assessment with biopsy or transurethral resection. Careful clinicopathological correlation, and sometimes additional immunohistochemistry or molecular analysis, may be needed to distinguish CG and CCEG from primary or secondary adenocarcinoma.

The malignant potential of CG, particularly the intestinal subtype, remains controversial. Case reports describe adenocarcinoma arising in areas of long-standing CG, but larger series have failed to show a consistent increase in cancer risk. More recently, genomic and transcriptomic studies have begun to explore shared pathways between CG and bladder carcinoma. There is a lack of long-term longitudinal clinical outcome data and conflicting evidence regarding malignant transformation.

In this study, we describe a single-institution experience with CG, cystitis cystica, and CCEG over a 10-year period. We report demographic data, clinical presentation, pathological features, management, and long-term outcomes, and we review the contemporary literature with particular attention to histology, immunohistochemistry, molecular findings, and malignant potential.

## Materials and methods

We performed a retrospective review of patients diagnosed with cystitis at Guy's Hospital, London, between 2013 and 2023. Our inclusion criteria included patients with cystitis glandularis, cystitis cystica, or cystitis cystica et glandularis. We used electronic patient histopathology records to help identify these patients. For each patient, we extracted basic demographic data, including age, sex, and ethnicity. We then went on to collect information on smoking status (where available), presenting symptoms, and indications for referral, as well as evidence of bladder outlet obstruction. Note was made of investigations and results, including imaging studies (ultrasound, CT urography, CT abdomen/pelvis, MRI), urinary cytology, and cystoscopic findings (including lesion location and extent). Histopathological diagnosis, including the presence of CG, intestinal-type metaplasia, cystitis cystica, and any associated urothelial carcinoma or other pathology, was recorded. Lastly, data regarding treatment (conservative management, transurethral resection, reconstructive surgery, cystectomy), follow-up duration, and surveillance cystoscopy, as well as development of recurrent lesions or malignancy during follow-up, were collected.

Bladder outlet obstruction was defined as poor urinary flow, elevated post‑void residual volume, and/or radiological evidence of obstruction such as hydronephrosis or ureteric dilatation in the presence of compatible symptoms. All histological diagnoses were made or confirmed by specialist uropathologists. CG was classified as conventional or intestinal type based on morphology. The presence of cystitis cystica, intestinal metaplasia, non-keratinizing squamous metaplasia, and coexisting urothelial carcinoma was recorded.

Descriptive statistics were used to summarize patient characteristics, clinical features, and outcomes. Continuous variables are presented as medians with interquartile ranges (IQR), and categorical variables as counts and percentages.

## Results

Patient characteristics

A total of 67 patients were identified (Table 1). The age range was 39-84 years, with a median age of 61 years (IQR: 54-69.5). The cohort was predominantly male - 57 patients (85.1%) were men and 10 (14.9%) were women. Ethnicity was recorded as White in 21 patients (31.3%), Asian in six (9.0%), Black in 14 (20.9%), and unknown in 26 (38.8%). Smoking status was available for 57 patients (85.1%). Of these, 21 (31.3%) were smokers and 36 (53.7%) non-smokers. Median follow-up was 9.2 years (IQR: 7-18), with a range from 2.2 to 17.8 years.

**Table 1:** Patient demographics and follow-up. Data are presented as n (%) for categorical variables and median (IQR) for continuous variables.

Variables	Values
Median age (years)	61 (IQR: 54-69.5)
Median follow-up (years)	9.2 (IQR: 7-18)
Male, n (%)	57 (85.1)
Female, n (%)	10 (14.9)
White, n (%)	21 (31.3)
Asian, n (%)	6 (9)
Black, n (%)	14 (20.9)
Unknown ethnicity, n (%)	26 (38.8)

Clinical presentation

Presenting complaints are summarized in Table 2. The most frequent presentation was lower urinary tract symptoms (LUTS), reported by 19 (28.4%) patients. Visible hematuria, with or without associated LUTS, was seen in 14 (20.9%) patients, and non-visible hematuria (with or without LUTS) in 11 (16.4%) patients. Five patients (7.5%) presented with flank or abdominal pain, and three (4.5%) were identified during routine surveillance for urothelial carcinoma. When hematuria was analyzed separately, visible hematuria at any time was documented in 28 (41.8%) patients, and non-visible hematuria in 32 (47.8%) patients. LUTS were present in 25 (37.3%) patients.

**Table 2:** Clinical presentation. LUTS: lower urinary tract symptoms; TCC: transitional cell carcinoma; VH: visible hematuria; NVH: non-visible hematuria

Variables	n (%)
LUTS	19 (28.4)
VH±LUTS	14 (20.9)
NVH±LUTS	11 (16.4)
Flank or abdominal pain	5 (7.5)
TCC follow-up presentation	3 (4.5)
Visible hematuria (any)	28 (41.8)
Non-visible hematuria (any)	32 (47.8)

Evidence of bladder outlet obstruction was identified in 10 patients (14.9%) based on symptoms, poor flow, and high post-void residuals, while 56 (83.6%) had no evidence of obstruction. A previous history of urothelial carcinoma (UC) was recorded in 13 patients (19.4%). Only one patient had a history of self-catheterization, and none had a permanent indwelling catheter.

Imaging and cytology

Imaging was available for 60 patients (89.6%). Of these, 35 (52.2%) underwent ultrasound of the kidneys, ureters, and bladder (USS KUB), 15 (22.4%) had CT urography (CTU), six (9.0%) had CT abdomen/pelvis (CT AP), and four (6.0%) patients underwent pelvic MRI.

Urinary cytology was available for 26 patients (38.8%). Seventeen (25.4%) demonstrated atypical or suspicious urothelial changes not diagnostic of carcinoma in situ, while nine (13.4%) showed no cytological abnormality.

Cystoscopic findings

Lesions were most frequently located at the bladder neck or trigone (42 cases, 62.7%). The lateral walls were involved in 14 cases (20.9%) and the posterior wall in two (3.0%). In nine (13.4%) patients, the site was not specified in the operative or endoscopy report. Lesions ranged from flat or slightly raised plaques to larger polypoid or papillary-appearing masses that were difficult to distinguish visually from urothelial carcinoma.

Histopathology

Histopathological findings are shown in Table 3. Cystitis cystica was present in 53 patients (79.1%). In six patients (9.0%), cystitis cystica was associated with urothelial carcinoma. Non-keratinizing squamous epithelium, with or without cystitis cystica, was found in three cases (4.5%), and cystitis cystica with focal intestinal metaplasia was identified in one patient (1.5%). Cystitis glandularis was present in 30 patients (44.8%), and absent in 37 (55.2%) patients. The intestinal-type variant of CG was identified in 16 patients (23.9%), while 51 (76.1%) did not show this pattern.

**Table 3:** Histopathology findings. TCC: transitional cell carcinoma

Variables	n (%)
Cystitis cystica only	53 (79.1)
Cystitis cystica+TCC	6 (9)
Non-keratinizing squamous±cystitis cystica	3 (4.5)
Cystitis cystica+focal intestinal metaplasia	1 (1.5)
Cystitis glandularis present	30 (44.8)
Intestinal type present	16 (23.9)

During follow-up, 39 patients (58.2%) underwent surveillance with flexible cystoscopy to assess for recurrence or progression. No case in this series demonstrated subsequent development of carcinoma arising from CG or CCEG during the follow-up period.

Interventions

Most patients were managed with endoscopic treatment, usually transurethral resection of the lesion, combined with treatment of underlying causes, such as infection or bladder outlet obstruction, where appropriate.

In patients with extensive disease involving the trigone and/or bladder neck, resection was sometimes staged to avoid excessive resection in a single sitting. Two patients in our cohort developed significant upper tract obstruction related to extensive trigonal disease and underwent robot-assisted bilateral ureteric reimplantation, with preservation of renal function. These cases illustrate the potential of CG and CCEG to cause clinically significant obstruction when involvement is extensive, and imaging in both demonstrated bilateral hydronephrosis pre-operatively. No patient in this cohort required cystectomy for CG or CCEG alone.

## Discussion

Cystitis glandularis (CG) and cystitis cystica et glandularis (CCEG) remain diagnostically and clinically challenging despite being benign entities. Their potential to mimic malignancy, both endoscopically and histologically, often leads to patient anxiety and occasionally to unnecessary aggressive management. Our 10-year experience, one of the largest contemporary single-institution series, reinforces the view that these are largely reactive, non-neoplastic conditions that rarely progress to cancer [1-3].

The demographic distribution in our series of predominantly male patients in their sixth decade closely mirrors previously published cohorts [18,19]. The most typical presentations were lower urinary tract symptoms and hematuria, and the lesions most often involved the bladder neck or trigone. This pattern is consistent with the concept that chronic irritation, urinary stasis, and turbulence at the dependent bladder base may drive the metaplastic response [20,21]. Roughly one in five of our patients had a prior history of urothelial carcinoma, which likely reflects a shared background of chronic inflammation rather than a causal relationship between CG and malignancy.

Histologically, cystitis cystica was present in nearly 80% of cases, cystitis glandularis in 45%, and the intestinal subtype in almost a quarter of cases. These proportions correlate with previous studies reporting intestinal metaplasia in 10-25% of cases [22,23]. Six patients had concurrent urothelial carcinoma, but none developed new carcinoma on follow-up, a median of 9.2 years, which is among the longest surveillance periods reported. This absence of malignant transformation supports the prevailing consensus that CG, even in its intestinal form, is a benign lesion. Similar findings have been described by Smith et al., who likewise found no progression to adenocarcinoma despite several years of follow-up [24].

Histological interpretation remains the cornerstone of diagnosis. In our cohort, the conventional type showed the expected glands lined by cuboidal or low columnar cells surrounded by residual urothelium, while the intestinal type exhibited tall columnar mucin-secreting cells and goblet cell differentiation [2]. These may be mistaken for intestinal‑type adenocarcinoma, particularly when mucus extravasation or irregular glandular patterns are present [25], underscoring the need to evaluate for absent cytological atypia, lack of desmoplasia, and non‑infiltrative growth [3,19]. Close clinicopathological discussion remains invaluable to avoid over-treatment.

The immunohistochemical and molecular features described in the literature complement these morphological findings. Conventional CG typically expresses CK7, GATA3, and p63, reflecting urothelial differentiation [20], whereas intestinal-type lesions show positivity for CDX2 and CK20 with reduced CK7 expression [7,21]. Molecular studies have revealed upregulation of proliferative and inflammatory pathways involving epidermal growth factor receptor (EGFR), androgen receptor (AR), and cyclin D1, in addition to increased chemokine signaling, suggesting that CG shares some cellular activity with inflammatory and neoplastic states but without clear evidence of oncogenic transformation [26]. These findings may help explain the documented coexistence of CG and carcinoma in some bladders without necessarily indicating biological progression from CG to malignancy [12].

Our clinical outcomes are in keeping with these observations. Most patients were managed conservatively or by transurethral resection of the lesion. The procedure was curative in almost all cases. Two patients with extensive trigonal disease and bilateral upper tract obstruction required robot-assisted bilateral ureteric reimplantation, both with excellent post-operative results and preserved renal function. These cases demonstrate that while CG is histologically benign, it can lead to significant anatomical obstruction when extensive. The robotic approach allowed precise resection and reconstruction with minimal morbidity, a strategy that should be considered in similarly complex cases [5].

Medical therapy has a limited role. Although isolated reports describe improvement with corticosteroids [11], cyclooxygenase-2 (COX-2) inhibitors [27], or intravesical curcumin, we found no need for these interventions [28]. Symptom control followed resection and management of underlying irritative factors, such as infection or obstruction. No patient in our series required cystectomy for CG alone, supporting the view that major surgery should be reserved for the rare, extensively fibrotic bladder causing severe storage symptoms or loss of capacity [29].

Follow-up in this series was selective and individualized. Patients with limited, completely resected lesions and no dysplasia were discharged after recovery. Those with more extensive or intestinal-type lesions, or with upper tract involvement, underwent periodic cystoscopic and imaging surveillance. No malignant transformation was observed in any subgroup. This experience supports a pragmatic, risk-adapted approach rather than routine long-term follow-up for all [13]. Our findings add to the body of evidence that cystitis glandularis and cystitis cystica et glandularis are reactive, benign proliferations rather than true premalignant lesions.

The link between cystitis glandularis and carcinoma, in our experience, seems to be coincidental rather than causal. Both tend to arise in chronically irritated bladders [4,6], and the two may share similar underlying conditions rather than one evolving into the other [24]. It is therefore important not to overinterpret their coexistence or treat every glandular lesion as a premalignant finding. Nonetheless, extensive disease can sometimes lead to clinically significant complications, such as bladder outlet or ureteric obstruction, which requires prompt recognition and, if necessary, reconstructive management [5,14,30].

Limitations

This study is limited by its retrospective nature and reliance on existing records, which inevitably introduces some data gaps. Ethnicity and smoking history were incompletely documented in a proportion of patients. Although follow-up was long, histological re-evaluation of earlier cases was not performed with uniform immunohistochemical or molecular testing, limiting direct correlation with newer biomarkers. Radiological and pathological findings were not reviewed again, but the results/records were relied upon. A degree of selection bias may have been present, as only histologically confirmed cases were included. Furthermore, as a single-center experience, our findings may reflect local referral patterns and practices. Nonetheless, the cohort size, long follow-up, and inclusion of both typical and intestinal variants provide a valuable addition to the existing literature.

## Conclusions

In our series, none of the patients developed carcinoma on follow-up, which supports the generally benign course reported elsewhere. In those with extensive trigonal disease, robotic ureteric reimplantation proved a practical way to relieve obstruction and protect renal function without the need for open surgery. Routine lifelong surveillance is unnecessary for most patients but should be considered in those with intestinal-type histology, dysplasia, or persistent symptoms.

Our 10-year institutional experience supports the view that cystitis glandularis, including its intestinal variant, is a benign and reactive condition with negligible malignant potential. While the disease can mimic malignancy and occasionally cause obstructive complications, conservative and minimally invasive surgical management yields excellent outcomes. Awareness of its natural history and careful histological assessment are essential to prevent overtreatment and to ensure appropriate care of patients with this uncommon but important entity.
